# Oromandibular Dystonia After Low-Dose Olanzapine Treatment in a Patient With Marchiafava-Bignami Disease With Demyelinating Lesions in the Corpus Callosum: A Case Report

**DOI:** 10.7759/cureus.52140

**Published:** 2024-01-11

**Authors:** Nagiho Tsukada, Naomichi Okamoto, Yuki Konishi, Atsuko Ikenouchi, Reiji Yoshimura

**Affiliations:** 1 Psychiatry, University of Occupational and Environmental Health, Kitakyushu, JPN

**Keywords:** delirium, olanzapine side effects, corpus callosum, marchiafava-bignami disease, oromandibular dystonia

## Abstract

Marchiafava-Bignami disease is a rare disorder characterized by demyelination and necrosis of the central nervous system. Dystonia is a movement disorder characterized by sustained or intermittent muscle contractions. Herein, we present the case of a patient with Marchiafava-Bignami disease who developed acute oromandibular dystonia after receiving a very low dose of olanzapine. He was a 60-year-old Japanese man who was diagnosed with demyelinating lesions in the corpus callosum associated with Marchiafava-Bignami disease. At one point, he became agitated at night and was administered olanzapine 2.5 mg, resulting in the onset of oromandibular dystonia; however, the symptoms disappeared upon discontinuation of the drug. Primary dystonia is believed to arise solely from abnormal basal ganglia function in the absence of apparent morphological changes, according to the traditional view. However, recent studies suggest the involvement of lesions beyond the basal ganglia and organic factors, including ultrastructural changes. Rare side effects that develop following small doses of olanzapine indicate that demyelinating lesions of the corpus callosum may be partially responsible for oromandibular dystonia. This case report supports previous reports that the corpus callosum is involved in dystonia and provides insights into the pathophysiology underlying oromandibular dystonia.

## Introduction

Marchiafava-Bignami disease is a rare demyelinating and necrotic disease of the central nervous system that most commonly occurs in chronic alcohol abusers and malnourished individuals [1]. Dystonia is a movement disorder characterized by sustained or intermittent muscle contractions that often result in abnormal repetitive movements, postures, or both [2]. Dystonia affecting the muscles involved in mastication, the tongue, and the pharynx is called oromandibular dystonia [3]. Oromandibular dystonia is caused by drugs/toxins, metabolic/endocrine factors, infections, and genetic factors [4]. Drug-induced oromandibular dystonia caused by dopamine receptor blockers is the most common form, in which the basal ganglia appear to be a core factor. The traditional view is that primary dystonia arises solely from abnormal basal ganglia function in the absence of apparent morphological changes [5]. However, recent advancements in imaging techniques have revealed the involvement of lesions outside the basal ganglia and ultrastructural changes. For example, diffusion tensor imaging shows reduced fractional anisotropy in the corpus callosum [4,6,7]. Here, we present a case of acute oromandibular dystonia after low-dose olanzapine treatment in a patient with Marchiafava-Bignami disease with demyelinating lesions of the corpus callosum.

## Case presentation

A 60-year-old Japanese man was admitted to the neurology department and referred to the psychiatric department for insomnia. He had no medical comorbidities, family history, or psychiatric disorders, but he had a history of alcohol consumption that commenced in his 20s, with significant daily abuse from the age of 50 years. After five years of alcohol abuse, he gradually became lethargic, with progressive anorexia and discomfort in his lower extremities.

**Figure 1:**
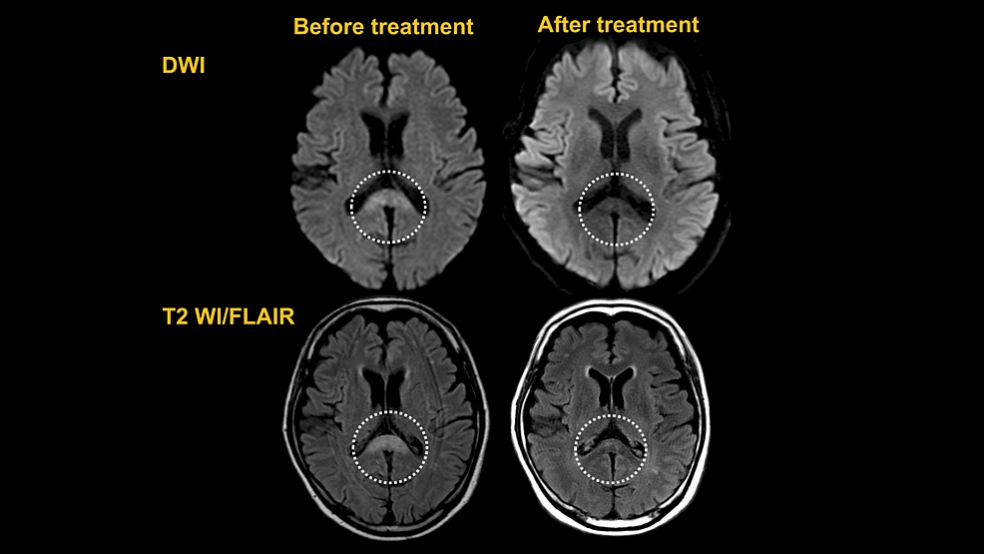
Head MRI (DWI and T2/WI FLAIR) Head MRI showing the changes that took place after treatment. The DWI and T2 WI/FLAIR signals show high density and swelling of the corpus callosum before treatment and atrophy and high-density improvement in the same area after treatment DWI: diffusion-weighted imaging; MRI: magnetic resonance imaging; T2 WI/FLAIR: T2-weighted/fluid-attenuated inversion recovery

**Figure 2:**
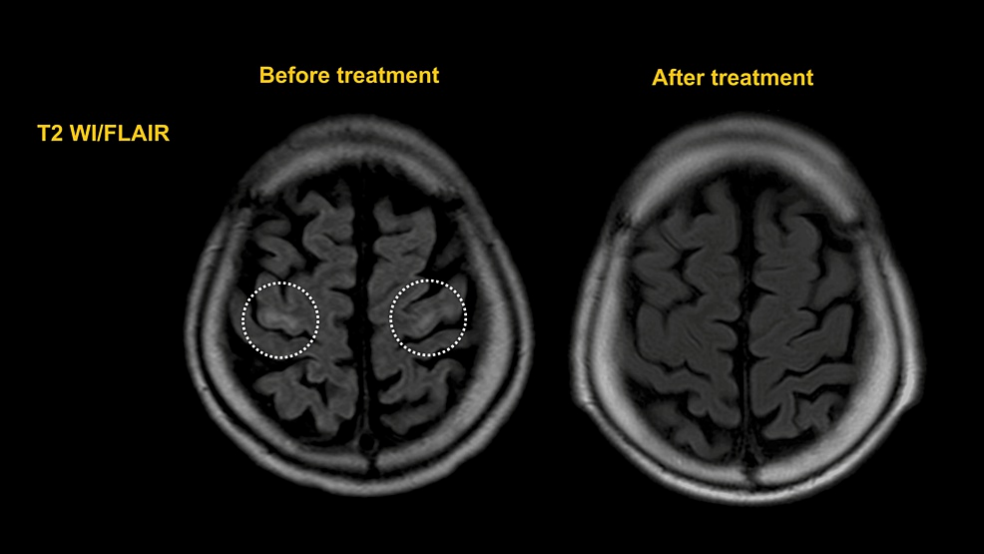
Head MRI (T2/WI FLAIR) Head MRI showing the changes that took place after treatment. T2 WI/FLAIR signals show high density of the precentral gyrus of the bilateral frontal lobes before treatment and its disappearance in the same area after treatment MRI: magnetic resonance imaging; T2 WI/FLAIR: T2-weighted/fluid-attenuated inversion recovery

He was admitted to the emergency department because of unsteadiness when walking that had been present for one month and an inability to stand up for one week. Wernicke's encephalopathy was suspected because of the loss of motivation, gait disturbance, and memory impairment. After treatment for dehydration and vitamin B complex supplementation, his motivation and memory improved, but he still experienced difficulty walking; therefore, he was transferred to the neurology department of our hospital for a thorough examination. Upon admission, his level of consciousness was normal (Glasgow Coma Scale: E4V4M6), and he presented with no vital abnormalities or cranial abnormalities, except for horizontal diplopia. Both of his lower extremities exhibited spastic paralysis and hypoesthesia. Laboratory investigations showed no increase in the inflammatory response, and there were no abnormal findings in electrolytes, liver function, blood glucose, endocrine hormones, ammonia, vitamins, or immunological tests. His urinalysis results were normal, and cerebrospinal fluid analysis using lumbar puncture ruled out inflammation or infection. The Mini-Mental State Examination (MMSE) showed moderate cognitive decline, with a score of 23/30 (place awareness, 3/5; computation, 3/5; and delayed regression, 0/3), and his Frontal Assessment Battery score was 17/18 (verbal fluency, 2/3). Electroencephalography recorded a background activity of 8-11 Hz, with no apparent epileptic discharges. We compared magnetic resonance imaging (MRI) scan of the patient's brain on admission to one taken at his previous hospital and another taken after one month of treatment. The scans revealed hyperintensity on diffusion-weighted imaging (DWI) and hyperintensity and swelling on T2-weighted/fluid-attenuated inversion recovery (T2 FLAIR) in the splenium of the corpus callosum, which improved and atrophied after treatment (Figure 1, circle). T2 WI/FLAIR signals revealed high density of the precentral gyrus of the bilateral frontal lobes before treatment, which disappeared in the same area after treatment (Figure 2, circle).

Therefore, Marchiafava-Bignami disease was diagnosed based on the patient's history, clinical symptoms, and imaging findings. The patient continued rehabilitation while receiving vitamin B complex (the total dose of thiamine chloride hydrochloride was 75 mg per day for one month), and his level of consciousness, cognitive dysfunction, and spastic paraplegia improved slowly. However, 11 days after admission, he started exhibiting intermittent confusion in speech and behavior, both during the day and night. During the night on the 12th day of admission, he became unstable and agitated, experiencing visual hallucinations, and attempted to climb over the bed fence. While the neurologist initially prescribed quetiapine 12.5 mg three times at night to address the symptoms, the patient experienced insomnia and was administered initial psychopharmacotherapy on the following day.

On examination, the patient remained restless and complained of visual hallucinations. Because his speech was disorganized and he was disoriented, he was judged to be delirious and was prescribed olanzapine 2.5 mg before sleeping. The next day, the patient showed involuntary opening of the mouth; when instructed to close his mouth, he was able to bring the mouth slightly towards closure, but had difficulty closing it completely and was unable to maintain closure. His mouth did not close even with sensory trick techniques, and he further showed difficulty with speech, chewing, and swallowing, and his mouth remained open almost all day. There were no other involuntary facial or trunk movements or worsening of his muscle tone. Based on the course of his symptoms, delirium and drug-induced dystonia were suspected, and olanzapine was discontinued. His drug-induced extrapyramidal symptoms scale score for dystonia was 4. Over approximately 10 days, the muscle tone around his mouth reduced, and oromandibular dystonia disappeared without the use of anticholinergic or other medications. Subsequently, his delirium improved, and his MMSE score improved to 28/30 points at the end of his five-week stay at our hospital. The patient was then able to walk using a walker, and he was transferred to a rehabilitation hospital. He experienced no neurological deterioration or recurrence of delirium or oromandibular dystonia after six months.

## Discussion

Herein, we reported a case of acute oromandibular dystonia after low-dose olanzapine treatment in a patient with Marchiafava-Bignami disease. This case has pathophysiological utility as it presents the possibility of the involvement of dopamine and the corpus callosum in the pathophysiology of oromandibular dystonia.

Marchiafava-Bignami disease is a rare demyelinating and necrotic disease of the central nervous system that most commonly occurs in chronic alcohol users or malnourished individuals [1]. The main pathophysiology is alcohol consumption, which results in thiamine deficiency and the inhibition of various metabolic pathways. Myelin synthesis and signal transduction are also impaired, resulting in a variety of neurological signs and symptoms. Dystonia is a movement disorder characterized by sustained or intermittent muscle contractions that often causes abnormal repetitive movements, postures, or both [2]. On the other hand, oromandibular dystonia is defined as any condition involving the masticatory, lingual, or pharyngeal muscles [3]. Idiopathic focal oromandibular dystonia is infrequent and has been observed in 3-5% of reported patients with dystonia [8].

Dopamine receptor blockers are the most common cause of drug-induced oromandibular dystonia [4]. Acute movement disorders, including dystonia, are believed to result from the blockade or denervation supersensitivity of dopamine receptors along nigrostriatal and mesocortical dopaminergic pathways [9]. These findings align with the empirical treatment approach, suggesting that dystonia tends to improve with the administration of dopaminergic and anticholinergic medications [4]. In this patient, dystonia appeared after the start of olanzapine treatment and improved after discontinuation; however, it did not appear with quetiapine, which has a weak dopamine 2 receptor-blocking effect. Therefore, the dopamine 2 receptor-blocking effect of olanzapine was likely involved. Notably, a positive correlation has been shown between the rate of binding to dopamine 2 receptors and the frequency of movement disorders [10]. However, the prevalence of acute dystonia is lower with the atypical antipsychotic olanzapine (1.6%) than with typical antipsychotics [11]. Furthermore, compared to previous reports on acute drug-induced dystonia [12,13], the present dose of 2.5 mg is extremely low.

In terms of organic diseases, a previous study showed that the lateral portion of the substantia nigra and the caudoventral portion of the putamen and pallidum are primarily involved with orolingual and head movement and that the basal ganglia in one cerebral hemisphere play a role in bilateral orofacial-lingual motor control [14]. Another study indicated that reduced excitability of cortical inhibitory interneurons or reduced facilitation of inhibitory interneurons from subcortical or other cortical structures plays a role in the pathogenesis [7]. The traditional view is that primary dystonia arises solely from abnormal basal ganglia function in the absence of apparent morphological changes [5]; however, recent improvements in imaging technology have indicated the involvement of lesions other than the basal ganglia and ultrastructural changes as organic factors in dystonia [4,6]. Mild neuronal loss related to neurofibrillary tangles has further been observed in the brainstem nuclei, and the corpus callosum has been found to have ultrastructural abnormalities and a decreased number of fibers [5,6]. Notably, a prior report showed that diffusion tensor imaging indicates decreased fractional anisotropy in the genu and body of the corpus callosum [5], the largest bundle of fibers that connects the cortical and subcortical regions of the brain [15]. Furthermore, the corpus callosum also interconnects both cerebral hemispheres, facilitating the functional integration of motor mechanisms [16]. A decreased number of fibers in the corpus callosum may result in altered motor cortex excitability and could be associated with dystonia [5].

Taken together, these findings suggest that acquired corpus callosum dysfunction may induce dystonia by affecting the function and excitability of cortical or subcortical-related neurons, combined with the dopamine 2 receptor-blocking effect of olanzapine. To the best of our knowledge, this is the first report of a patient with Marchiafava-Bignami disease with demyelinating lesions of the corpus callosum who showed oromandibular dystonia after low-dose olanzapine treatment.

## Conclusions

Herein, we report a case of oromandibular dystonia after low-dose olanzapine treatment in a patient with Marchiafava-Bignami disease and demyelinating lesions in the corpus callosum. The rare side effects of small doses of olanzapine indicate that demyelinating lesions of the corpus callosum may be partially responsible for oromandibular dystonia. This case supports previous reports that the corpus callosum facilitates the integration of the motor mechanism, is involved in dystonia, and provides new insights into the pathophysiology of oromandibular dystonia.
